# Two-Dimensional Estrone-Imprinted System on a Self-Assembled Monolayer

**DOI:** 10.3390/polym16142035

**Published:** 2024-07-17

**Authors:** Min Jae Shin, Jae Sup Shin

**Affiliations:** 1 Department of Chemical and Biological Engineering, Andong National University, Andong 36729, Gyeongbuk, Republic of Korea; newminj@gmail.com; 2 Department of Chemistry, Chungbuk National University, Cheongju 28644, Chungbuk, Republic of Korea

**Keywords:** estrone, self-assembled monolayer, selectivity factor, molecular imprinting

## Abstract

In this study, a thin poly (methyl methacrylate) coating was formed on a self-assembled monolayer formed on a gold plate after chemically binding estrone. Subsequently, the estrone molecules were hydrolyzed and extracted using a solvent to form a molecular-imprinted system. The estrone-imprinted gold plate was then used as a working electrode to measure the estrone recognition ability through electrochemical methods. The recognition ability of this working electrode was evaluated for similar compounds. The selectivity factors for the seven estrone analogs were measured, and these values ranged from 0.19 to 0.67. According to the experimental results, the estrone-imprinted system showed good differentiation of estrone from other estrone analogs. Comparing these selectivity factors with those of a previous study on a cholesterol-imprinted system, the relative molecular size difference between the target molecule and similar molecules had a significant impact on the selectivity factor.

## 1. Introduction

Molecular imprinting is a technique that forms a mold of a specific molecule and uses it as a tool to recognize that molecule, thus mimicking biological systems; it has distinct advantages and disadvantages [1,2,3,4]. One advantage of molecular imprinting is the ability to overcome the various limitations of biological systems. For example, molecular imprinting allows the use of various organic solvents, operates over a wide pH range, and exhibits a broad range of operating temperatures. Another significant advantage is that these molecular-imprinted systems can be produced using inexpensive materials and simple methods. However, one disadvantage is that these systems have lower selectivity than biological systems. Despite their lower performance, the numerous clear advantages of molecular-imprinted systems have led to continuous research and development in this field [5,6,7,8,9].

Molecular imprinted systems are generally based on polymers with three-dimensional network structures. Typically, a target molecule is mixed with a monomer to form a three-dimensional network structure, and polymerization proceeds to synthesize a polymer mass in which the target molecule is evenly distributed within the three-dimensional network structure. This polymer is then finely ground into a very fine powder. When the target molecules located on the surface are extracted, the spots from which they were removed can be used as molds to recognize the target molecule [10,11,12,13,14].

Traditional molecular imprinting is typically performed using polymers with a three-dimensional network structure. However, two-dimensional (2D) molecular imprinting conducted on the surfaces of 2D materials, such as graphene [15,16] or very thin polymer films [17,18,19], has been introduced. In traditional molecular imprinting, a polymer with a three-dimensional network structure that includes the target compound is formed. This polymer is then finely ground to expose the target compound on the surface, and the target compound molecules are extracted using a solvent to create molecular recognition sites. Therefore, the depths at these sites vary widely and are random. In contrast, two-dimensional molecular imprinting allows control of the position where the target compound will be placed, and thus, the depth of the formed sites can be somewhat controlled. This results in more uniform molecular-imprinted sites, which can enhance the recognition ability. Moreover, two-dimensional molecular imprinting makes electrode fabrication much easier, thereby greatly benefiting sensor production. Measuring the molecular recognition ability of a molecular imprinting site is not easy, but one of the simplest methods is to use electrochemical techniques. To use such electrochemical methods, it must be possible to form an electrode using the material that has undergone molecular imprinting, and a 2D system allows for very easy access to electrode formation.

We attempted to expand the field of molecular imprinting technology by applying this technique on a self-assembled monolayer (SAM) for the first time. This technique was demonstrated to be feasible on a SAM [20,21]. Although molecular imprinting on the SAM surface is possible and has shown some ability, the formed sites do not have a great ability to discriminate between molecules, and this ability gradually decreases over time. It is believed that the reason for the decrease in discriminatory ability over time is related to the movement of the molecules forming the SAM on the gold surface [20]. To reduce the movement of molecules on the SAM, we synthesized a compound where a single molecule has two thiol groups that contact the gold plate. Using this compound in experiments similar to the previous ones, we were able to solve the issue of the decrease in discriminatory ability over time. However, the ability to discriminate between different molecules was not significantly improved [21].

To improve the performance of the research results obtained on a SAM, we published a study that involved coating the SAM with a very thin polymer film to make the interface of the molecular-imprinted system more distinct [22,23]. In this study, cholesterol, which is a crucial indicator of blood health status, was the target compound. In this system, after chemically binding cholesterol to the SAM, the SAM was covered with a very thin layer of poly (methyl methacrylate) (PMMA), and the bound cholesterol was hydrolyzed and removed to produce the molecular-imprinted system. These results confirm that this system can effectively discriminate between cholesterol and its analogous substance, cholic acid [22]. Furthermore, the selectivity factors for various cholesterol analogs were determined to assess how well the system differentiated these substances. The selectivity factors ranged from 0.042 to 0.56, showing significant differences depending on molecular shape [23].

In this study, estrone, an analog of cholesterol, was used as the target compound to construct a molecular-imprinted system similar to that used in the previous study, and selectivity factors for analogous substances were obtained. These selectivity values were then compared with those from a previous study to examine the impact of molecular shape on the selectivity factors.

## 2. Materials and Methods

### 2.1. Materials and Instruments

PMMA (MW 350,000), estrone, estradiol, cholesterol, deoxycholic acid, cholic acid, testosterone, β-estradiol 17-acetate, testosterone propanoate, benzenethiol, 4-mercaptophenol, triethylamine, sodium perchlorate, potassium ferricyanide [K_3_Fe(CN)_6_], pyridine, phosgene, and tetrahydrofuran (THF) were purchased from Aldrich (St. Louis, MO, USA). The circular gold plate used as the working electrode was custom-made in a local goldsmith shop. The gold plate has a diameter of 1.0 cm and a thickness of 0.50 mm, with a 2.0 cm lead wire attached, with both sides of the plate used in the experiments. Before use, the gold plate was cleaned with a piranha solution prepared by mixing 90 mL of concentrated sulfuric acid with 30 mL of 30 wt% H_2_O_2_. The cleaning process involved immersing the gold plate in piranha solution for 15 min, followed by immersion in distilled water for 10 min, and finally rinsing with distilled water. Cyclic voltammograms were obtained using an Ivium potentiostat (Ivium Technologies, Eindhoven, The Netherlands), spin coating was performed using an ACE-200 spin coater (Dong Ah Trade Co., Seoul, Republic of Korea), and the coating thickness was measured using a Horiba Uvisel 2 Ellipsometer (Kyoto, Japan). Scanning electron microscopy (SEM) was performed using a Hitachi S-5200 scanning electron microscope, Chiyoda, Japan.

### 2.2. Synthesis of Estronyl Chloroformate

Estronyl chloroformate was synthesized using a previously reported method [24]. ^1^H NMR (500 MHz, CDCl_3_) δ 0.82 (s, 3H), 1.35 (m, 3H), 1.47 (m, 2H), 1.55 (m, 1H), 1.74 (m, 1H), 1.92 (m, 2H), 2.05 (m, 2H), 2.29 (m, 1H), 2.42 (m, 1H), 2.71 (m, 2H), 6.86 (m, 1H), 6.93 (m, 1H), and 7.31 (m, 1H).

### 2.3. Formation of a SAM on the Surface of the Gold Plate

A solution of the thiol compounds was prepared by dissolving 25.2 mg (0.20 mmol) of 4-mercaptophenol and 198 mg (1.80 mmol) of benzenethiol in 100 mL of ethanol. The prepared gold plate was immersed in this solution for 12 h to form a SAM on the gold surface. The gold plate was then immersed in 100 mL of ethanol for 30 min to remove the overcoated thiol compounds and dried under vacuum for 1 h. All processes were performed with the gold plate suspended vertically, and all reactions occurred simultaneously on both sides of the plate.

### 2.4. Reaction of Estronyl Chloroformate with 4-Mercaptophenol on the SAM

The gold plate with the SAM formed in the previous step was immersed in 30 mL of THF solution containing 1.0 mL of triethylamine. A solution of 1.30 g (3.73 mmol) of estronyl chloroformate dissolved in 15 mL THF was added to this mixture. The reaction mixture was then stirred for 6 h. After the reaction, the gold plate was rinsed with THF.

### 2.5. Purification of PMMA and Spin Coating of PMMA on the Gold Plate

To remove the additives and very low-molecular-weight PMMA from the purchased PMMA, 1.0 g of PMMA was dissolved in acetone (20 mL) and precipitated dropwise into methanol (100 mL) under stirring. This process was repeated two additional times. The resulting PMMA precipitate was collected and dried under a vacuum for 10 h. The PMMA solution for spin coating was prepared with 0.30 wt% PMMA in toluene, and spin coating was performed at 6000 rpm for 80 s. The PMMA-coated gold plate was dried under vacuum for 5 h before being used in subsequent experiments.

### 2.6. Formation of Estrone-Imprinted Sites on the Coating Surface

To hydrolyze the carbonate group linking estrone and the SAM, the gold plate was placed in a 1.0 M NaOH methanol solution and refluxed for 3 h. The plate was then rinsed five times with distilled water. The hydrolyzed estrone was extracted using hexane, followed by extraction with methanol, and the extraction process with hexane and methanol was repeated.

### 2.7. Electrochemical Measurements

The recognition ability of the molecular-imprinted system was measured electrochemically. The electrochemical system was configured as a three-electrode system in a 50 mL cell, with the estrone-imprinted gold plate as the working electrode, an Ag/AgCl (3 M KCl) electrode as the reference electrode, and a 10 cm platinum wire as the counter electrode. The redox reaction of potassium ferricyanide was used as a background reaction. A solution of 0.0495 g (0.150 mmol) of potassium ferricyanide and 0.1838 g (1.50 mmol) of sodium perchlorate was prepared in a solvent containing 15 mL of ethanol and 15 mL of H_2_O, creating a 5.0 mM potassium ferricyanide and 50 mM sodium perchlorate solution. Cyclic voltammograms were measured at a scan rate of 50 mV/s between –0.5 V and 0.5 V. The concentration of the compound to be measured was increased by 5.0 μM increments, and the maximum current in the background oxidation reaction was recorded to measure how the increase in concentration affected the maximum current.

### 2.8. Formation of a Non-Imprinted Electrode

For comparative evaluation, a non-imprinted electrode was prepared following the same process as that for the estrone-imprinted electrode, except the reactions with estronyl chloroformate and hydrolysis of the carbonate group were omitted.

## 3. Results and Discussion

### 3.1. Formation of Estrone-Imprinted Sites on the SAM

In this study, a molecular-imprinted system was fabricated using a SAM. First, we formed a SAM on a gold plate prepared using 4-mercaptophenol and benzenethiol. Based on previous studies of cholesterol-imprinted systems, a ratio of 4-mercaptophenol to benzenethiol of 1:9 was used. The hydroxy group of 4-mercaptophenol reacts with estronyl chloroformate, forming the imprinted site. The surface of the gold plate was then spin-coated with a 0.30 wt% PMMA solution at 6000 rpm for 80 s to form a very thin PMMA film. A very low-concentration PMMA solution and high-speed spin coating were used to minimize the thickness of the resulting film. The PMMA used in this study was obtained from Sigma Aldrich (St. Louis, MO, USA) and had an Mw of 350,000, which was purified before use. The purification process involved dissolving PMMA in acetone, precipitating it in methanol dropwise with stirring, and filtering and collecting the precipitate. This process was repeated thrice. The Mw of the obtained PMMA was determined to be 378,000 by gel permeation chromatography. The increase in Mw is considered to be due to the removal of the lower molecular weight portion of PMMA during the purification process. The thickness of the final film measured after drying under vacuum was 3.3 ± 0.2 nm. This thickness is very significant because if it becomes much larger, it will be impossible for the estrone molecules bound to the SAM to be exposed, making the subsequent hydrolysis and extraction steps difficult to proceed with. If it becomes lower than this, the formation of the imprinted site will not be distinct, reducing the ability to discriminate between different molecules. The adjustment of this thickness was made possible by controlling the speed and timing of the spin coating. This adjustment is well explained in our previously published paper [20,21].

**Figure 1:**
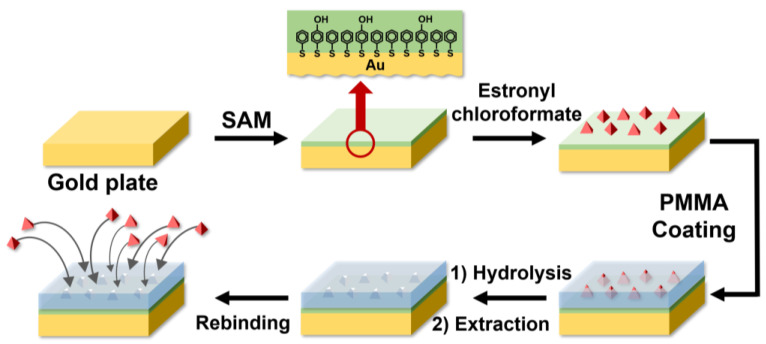
A schematic diagram of the estrone imprinting process. The red arrow signifies magnification.

Estrone linked through a carbonate functional group was hydrolyzed, and a solvent was used to extract estrone and form the estrone-imprinted site. After the formation of the estrone-imprinted site, the thickness of the film was 3.5 ± 0.4 nm. It was determined that there was no significant change in the thickness of the film during the formation of the estrone-imprinted site. However, the margin of error after the formation of the estrone-imprinted site increased by about twice compared to before the formation. This suggests that the roughness of the film surface had increased. The formation process of the estrone-imprinted site is illustrated in Figure 1.

As shown in Figure 1, the first step involves the formation of a SAM on a gold plate using 4-mercaptophenol and benzenethiol. In the second step, estronyl chloroformate reacts with the SAM. The third step consists of coating with PMMA. In the fourth step, molecular-imprinted sites are formed through hydrolysis and extraction. The fifth step is rebinding with estrone.

**Figure 2:**
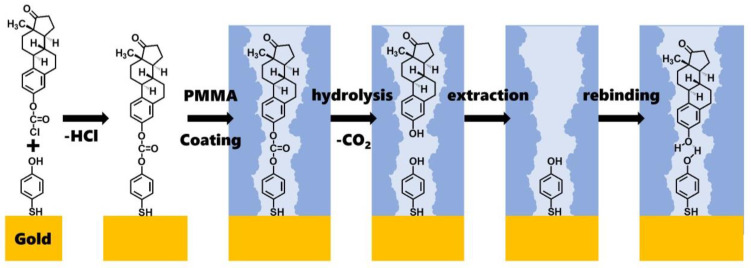
The detailed chemical reactions in this molecular imprinting process.

The detailed chemical reactions that occurred during molecular imprinting are shown in Figure 2. The first step involves the reaction between estronyl chloroformate and the hydroxy group of 4-mercaptophenol to form a carbonate group. The second step is coating it with PMMA. The third step consists of the hydrolysis of the carbonate group by NaOH, causing estrone to detach. The fourth step is the extraction of the detached estrone using hexane and methanol. This results in the formation of molecular-imprinted sites. The fifth step shows the process of estrone rebinding to the molecular-imprinted sites. Here, hydrogen bonding between the hydroxy groups of estrone and 4-mercaptophenol plays an important role in the rebinding process.

One significant difference between the molecular imprinting process shown in Figure 1 and the conventional molecular imprinting process is that, in our method, we control the depth of the molecular-imprinted sites to create sites with relatively uniform depth. In conventional molecular imprinting, the target compound and monomer are combined and polymerized, and the resulting bulk polymer is finely ground into a powder. This process exposes many target compounds on the surface of the polymer powder. When these target compounds are extracted with a solvent, the sites from which they are removed become the molecular-imprinted sites. However, the depths of these sites were determined randomly, and the proportion of practically useful molecular-imprinted sites was very low. In contrast, our study did not involve grinding the polymer as in the conventional process, and we could control and determine the depth of the sites, offering a significant advantage.

We obtained SEM images to examine the surface morphology of the electrode imprinted with estrone. The results are shown in Figure S1 of the Supplementary Materials. Additionally, to compare this with the surface of a non-imprinted electrode, we obtained SEM images of the non-imprinted electrode’s surface, shown in Figure S2.

Upon examining the results in Figures S1 and S2, it was very difficult to discern the difference between the estrone-imprinted surface and the non-imprinted surface in the SEM images. The attempt to obtain SEM images was made three times with different samples, all showing similar results. Despite trying various magnifications, it was impossible to detect any differences between the estrone-imprinted and non-imprinted surfaces. Moreover, cracks can be observed in both Figures S1 and S2, which are believed to have formed during the vacuum drying process after coating.

### 3.2. Recognition Ability Estimation of the Estrone-Imprinted Sites

**Figure 3:**
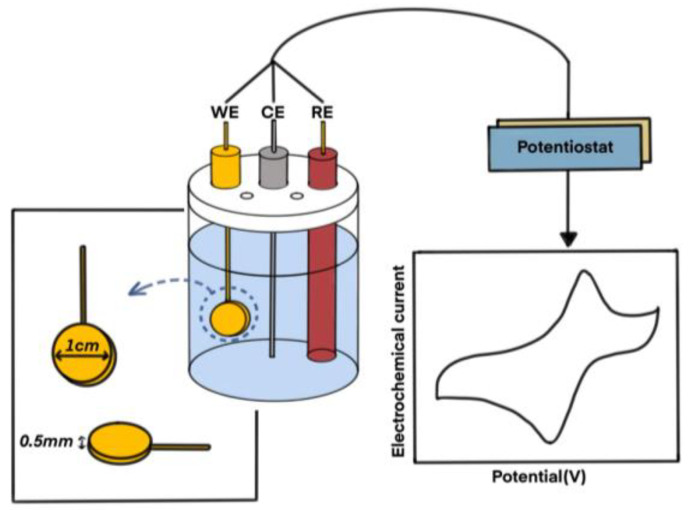
Schematic diagram of the redox reaction apparatus and process conducted in this study. WE: working electrode, CE: counter electrode, RE: reference electrode.

The ability of the estrone-imprinted site to recognize estrone was measured using an electrochemical method. First, a three-electrode system was configured, consisting of a reference electrode made of Ag/AgCl, a counter electrode made of platinum wire, and a working electrode made of the gold plate imprinted with estrone, developed in this study. The configuration of this three-electrode system is shown in Figure 3.

In this configured system, the redox reaction of potassium ferricyanide was carried out as a background reaction. Then, by adding various compounds, including estrone, to this background reaction, the decrease in maximum current was measured to determine how well each compound fits into the formed estrone-imprinted site. An example of the actual data of the cyclic voltammogram of the redox reaction measured using a potentiostat is shown in Figure S3 (Supplementary Materials).

**Figure 4:**
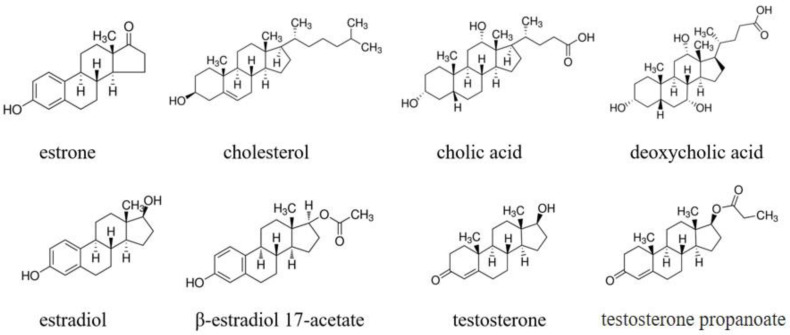
Chemical structures of estrone analogs used in this study.

The recognition ability of the estrone-imprinted system was evaluated by testing its ability to discriminate between estrone and seven structurally similar molecules. The molecular structures of the eight compounds, including estrone, are shown in Figure 4.

**Figure 5:**
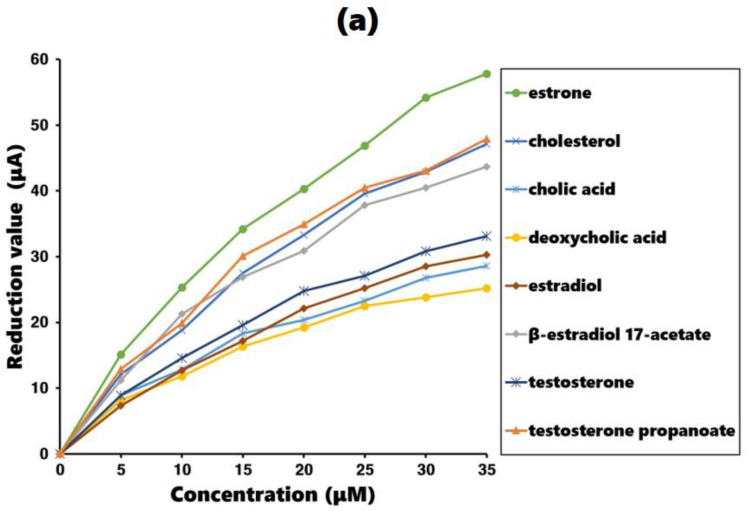

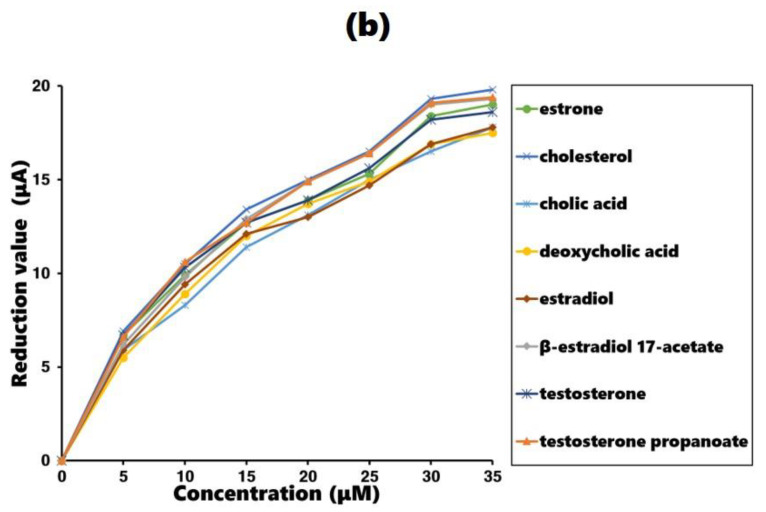
Current reduction value in the cyclic voltammogram with estrone and estrone analog addition: (**a**) estrone-imprinted electrode and (**b**) non-imprinted electrode.

The measurements were conducted using an estrone-imprinted gold plate as the working electrode. The maximum current was measured during background oxidation reaction. The experimental process involved adding estrone or estrone analogs to the test solution in 5 µM increments and recording the reduced current due to the additions. The additions continued until a concentration of 35 µM was reached, resulting in a continuous decrease in the maximum current. The better the fit of the added molecule to the estrone-imprinted electrode, the greater the decrease in the maximum current, which serves as an indicator of how well the added molecule fits the imprinted site. The results are shown in Figure 5a, with comparison results obtained using a non-imprinted gold plate as the working electrode shown in Figure 5b.

The reproducibility and error of these experiments were determined from the results of three replicates. If the results of these three experiments fell within a ±7% range of the average value, the average value was confirmed as the experimental result. If the results fell outside this range, two additional experiments were conducted, and among the total of five experimental results, the highest and lowest values were excluded. The average of the remaining three data points was then used as the experimental result. By following this procedure, the error range of all data was within the ±7% range.

Examining the results in Figure 5a, the estrone-imprinted electrode shows a much larger decrease in current for estrone than its analogs. This indicates that the binding sites on the estrone-imprinted electrode are most suitable for estrone, demonstrating a clear imprinting effect. Among the estrone analogs, testosterone propanoate, cholesterol, and β-estradiol 17-acetate exhibited similar reductions, whereas the other four analogs showed relatively larger reductions. A common feature of the first three analogs is that they all have side chains with hydrophobic ends. In contrast, the remaining four analogs lacked side chains or had side chains containing hydrophilic functional groups at their ends. For comparison, the results obtained using the non-imprinted electrode are shown in Figure 5b, where all eight compounds produced similar values.

To further investigate the effects of the compounds on imprinted sites, the results were qualitatively analyzed. Using the reduced current values at 30 µM for each compound, the imprinting factor and selectivity factor were estimated. The imprinting factor was calculated using the following formula:

Imprinting factor = Red_MI_/Red_NI_

where Red_MI_ is the reduced current obtained with the estrone-imprinted electrode and Red_NI_ is the reduced current obtained with the non-imprinted electrode. The selectivity factor was calculated using the following formula:

Selectivity factor = ΔRed_analog_/ΔRed_estrone_

where ΔRed_analog_ is the difference between Red_MI_ and Red_NI_ for the estrone analog, and ΔRed_estrone_ is the difference between Red_MI_ and Red_NI_ for estrone. The selectivity factor indicates the extent to which the system differentiates estrone from its analogs. The imprinting and selectivity factors obtained in this study are listed in Table 1.

Table 1 shows that estrone had the highest ΔRed value of 35.8. Among the estrone analogs, deoxycholic acid had the lowest value of 6.9, whereas testosterone propanoate had the highest value of 24.1. These values indicated that the estrone-imprinted electrode functioned well. Consequently, the imprinting factor was the highest for estrone at 2.95, with those of estrone analogs ranging from 1.41 to 2.27. The selectivity factor, which indicates how well the estrone-imprinted electrode differentiates between estrone and its analogs, ranged from 0.19 to 0.67. For comparison, the selectivity factors obtained using a cholesterol-imprinted electrode in a previous study are listed in Table 1. The previous selectivity factors were significantly lower, indicating that the cholesterol-imprinted electrode more effectively discriminated than other molecules. A previous study concluded that smaller molecules, such as estrone, estradiol, and testosterone, or molecules with hydrophilic functional groups at their ends were better discriminated. In this study, molecules that were relatively small or had hydrophilic functional groups at their ends were better discriminated, whereas those with side chains and no hydrophilic functional groups, such as cholesterol, β-estradiol 17-acetate, and testosterone propanoate, were more poorly discriminated. The relative difference in the selectivity factors between the previous and current studies suggests that imprinting with larger molecules allows for better discrimination of smaller molecules.

In conclusion, for future attempts to develop experimental designs similar to those in this study, selecting target compounds with relatively large molecular shapes is recommended. This is advantageous if the compounds intended for differentiation have relatively smaller molecular shapes than the target compound.

## 4. Conclusions

In this study, a molecular-imprinted system was developed using a very thin polymer film with estrone as the target compound. A SAM was formed on a gold electrode. Estrone was bonded to the SAM, and a very thin polymer film was placed on top of the SAM to complete the system. After hydrolysis, the bonded estrone was extracted with a solvent to form a molecular-imprinted site. The ability to recognize estrone at the molecular-imprinted site was measured using the maximum current of the background oxidation reaction. This result was compared with those of seven analog compounds to measure the selectivity factor. The estrone-imprinted electrode exhibited the greatest decrease in the current for estrone, indicating its selectivity. The selectivity factors ranged from 0.19 to 0.67. Compared to the values obtained in a previous study on a cholesterol-imprinted electrode, these values were somewhat higher. This difference is likely because estrone, the target compound in this study, is smaller than cholesterol, the target compound in the previous study.

## Figures and Tables

**Table 1:** The imprinting and selectivity factors for estrone and its analogs.

	Red_MI_	Red_NI_	ΔRed	Imprinting Factor	Selectivity Factor	The Other Selectivity Factor *
estrone	54.2	18.4	35.8	2.95	1	0.24
cholesterol	42.9	19.3	23.6	2.22	0.66	1
cholic acid	26.8	16.5	10.3	1.61	0.29	0.042
deoxycholic acid	23.8	16.9	6.9	1.41	0.19	0.12
estradiol	28.5	16.9	11.6	1.69	0.32	0.24
β-estradiol 17-acetate	40.5	19.1	21.4	2.12	0.60	0.38
testosterone	30.8	18.2	12.6	1.69	0.35	0.25
testosterone propanoate	43.1	19.0	24.1	2.27	0.67	0.56

* This value was obtained by using a cholesterol-imprinted electrode in a previous study [23].

## Data Availability

The original contributions presented in the study are included in the article, further inquiries can be directed to the corresponding authors.

## Supplementary Materials

The following supporting information can be downloaded at: https://www.mdpi.com/article/10.3390/polym16142035/s1, Figure S1. SEM image of the estrone-imprinted electrode surface. Figure S2. SEM image of the non-imprinted electrode surface. Figure S3. Cyclic voltammogram data at estrone concentrations of 0 µM and 35 µM.
