# Supplementary material for: Two-Dimensional Estrone-Imprinted System on a Self-Assembled Monolayer
Source: Polymers (Basel). 2024 Jul 17;16(14):2035. doi: 10.3390/polym16142035 (PMC11280610; doi:10.3390/polym16142035)
Supplement: Supplementary file 1 [file polymers-16-02035-s001.zip › polymers-3073599-supplementary.pdf]

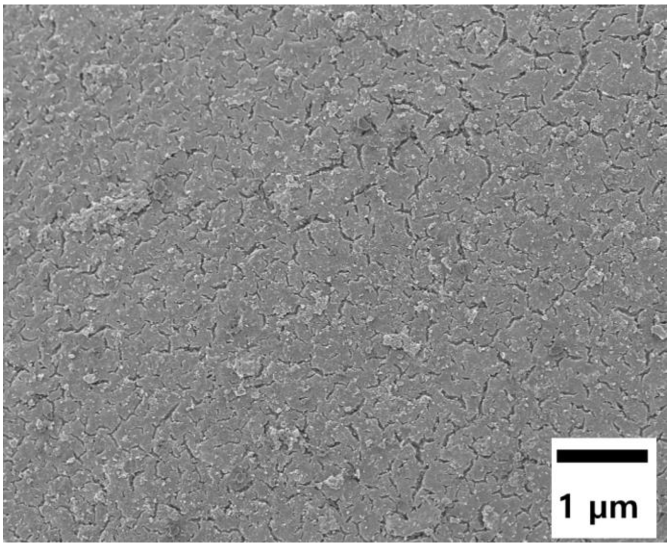

Figure S1. SEM image of the estrone-imprinted electrode surface.

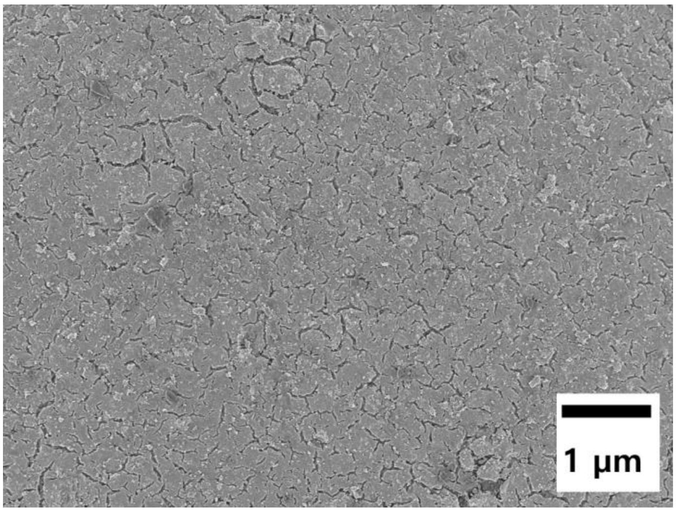

Figure S2. SEM image of the non-imprinted electrode surface.

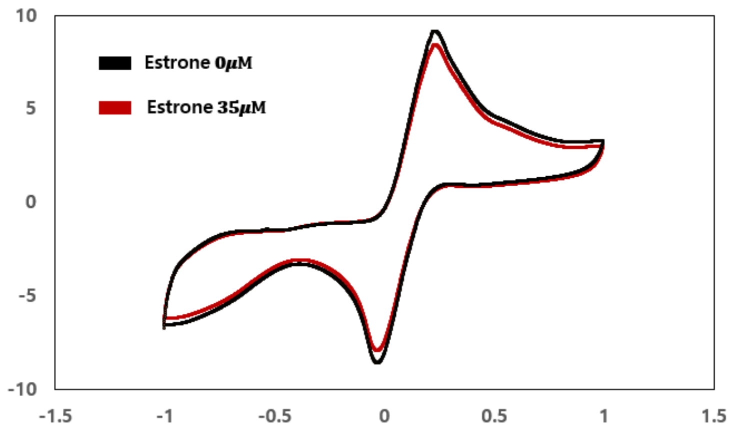

Figure S3. Cyclic voltammogram data at estrone concentrations of 0 μM and 35 μM.
